# *Helicobacter pylori vacA*, *cagA* and *iceA* genotypes in dyspeptic patients from southwestern region, Saudi Arabia: distribution and association with clinical outcomes and histopathological changes

**DOI:** 10.1186/s12876-019-0934-z

**Published:** 2019-01-25

**Authors:** Mohammed Akeel, Atef Shehata, Ahmed Elhafey, Erwa Elmakki, Thanaa Aboshouk, Hussein Ageely, Mohammed Mahfouz

**Affiliations:** 1 Department of Anatomy, Faculty of Medicine, Jazan University, Jazan, Kingdom of Saudi Arabia; 2 Department of Microbiology and Immunology, Faculty of Medicine, Jazan University, Jazan, Kingdom of Saudi Arabia; 3 Department of Pathology, Faculty of Medicine, Jazan University, Jazan, Kingdom of Saudi Arabia; 4 Department of Internal Medicine, Faculty of Medicine, Jazan University, Jazan, Kingdom of Saudi Arabia; 5 Department of Biochemistry, Faculty of Medicine, Jazan University, Jazan, Kingdom of Saudi Arabia; 6 Department of Family and Community Medicine, Faculty of Medicine, Jazan University, Jazan, Kingdom of Saudi Arabia; 7 Department of Microbiology and Immunology, Faculty of Medicine, Suez Canal University, Ismailia, Egypt; 8 Department of Pathology, Faculty of Medicine, Al-Azhar University, Cairo, Egypt

**Keywords:** *H. pylori*, PCR, Virulence genes, Genotype, Gastritis, Gastric ulcer

## Abstract

**Background:**

The aim of this study was to identify the common *H. pylori* virulence genes among dyspeptic Southwestern Saudi patients and their association with clinical outcomes and histopathological findings to help practitioners and researchers in the region for better management of infections caused by such bacteria.

**Methods:**

Four hundred two gastric biopsy specimens were analyzed using histopathological examination and real time-PCR. The positive 187 specimens by RT-PCR were genotyped using PCR targeting *cagA*, *vacA* and *iceA* genes.

**Results:**

One hundred twenty-eight gastric biopsy specimens were positive in genotyping PCRs. The *cagA*, *vacA*, *iceA*1 and *iceA*2 genes were detected in rates of 49.2% (63/128), 100%(128/128), 42.2% (54/128), 32.8% (42/128), respectively. The *vacA s1as1bm2* subtype was the highest 23.4% (30/128), followed by *m2* and *s1a1b* subtypes which were equally detected [16.4% (21/128) for each]. The *iceA* genes were significantly associated with gastritis and gastric ulcer. Overall, *vacA* genotypes were significantly associated with gastritis, gastric and duodenal ulcers. The *vacA* subtypes: *s1as1bm2*, *s1a1b* and *s2 m2* showed chronic active gastritis in percentages of 90.0, 81, and 84.2%, respectively. All *vacA* mixed genotypes showed chronic active gastritis.

**Conclusions:**

*H. pylori* virulence genes are highly prevalent and diverse among patients with dyspepsia in Southwestern region of Saudi Arabia. The *iceA* genes and the different *vacA* subtypes are significantly associated with the clinical outcomes and histopathological changes especially chronic active gastritis.

## Background

The World Health Organization (WHO) considers *H. pylori* as a bacterial carcinogen [1]. It has been reported that infection with *H. pylori* accounts for 75% of non-cardia gastric malignancy worldwide [2]. The *H. pylori*-induced gastritis can lead to atrophic gastritis, which in turn may progress to intestinal metaplasia, dysplasia and neoplasia, gastric adenocarcinoma and mucosa-associated lymphoid tissue lymphoma (MALT) [3, 4]. The mechanism of pathogenic effect of *H. pylori* is unclear, but it may be related to complex host bacterial interactions triggered by virulence genes. These effects may also be enhanced by the invasiveness of *H. pylori* [5]. The variation in the clinical outcomes may be attributed to the considerable genetic variation exists between strains of *H. pylori* [6, 7]_**.**_ The major *H. pylori* virulence genes are vacuolating cytotoxin A (*vacA*) and cytotoxin associated antigen A (*cagA*), and genes induced by contact with gastric epithelium (*ice* genes), which are of two types, *iceA*1 and *iceA*2 [8]. The *vacA* gene has many alleles such as *vacA s1*, *s2*, *m1* and *m2* [9]. The *cagA* gene is not present in all *H. pylori* strains, but is associated with clinical outcomes such as gastritis and peptic ulcer disease (PUD) as well as higher risk of occurrence of gastric carcinoma. [10, 11]. The *vacA* gene is found in all *H. pylori* strains, and some of its subtypes are associated with chronic inflammation of gastric mucosa and development of PUD [12]. This pathogenicity island is usually absent in *H. pylori* strains isolated from persons who are carriers of *H. pylori,* but are asymptomatic [13]. An important study on Saudi patients, which was conducted to detect the presence of *cagA*, *iceA*1, and *iceA*2 virulence genes in *H. pylori* from gastric biopsies, has reported a correlation between these genotypes and the development of PUD and gastritis [14]. A recent study in the Kingdom of Saudi Arabia has reported a high prevalence rate of *H. pylori* among dyspeptic patients and a strong correlation with duodenal ulcer [15]. Some studies have shown that the occurrence of gastric cancer is independent of *cagA* status and of other virulence factors (*cagE*, *cagT*, *vacA*, *babA* and *hrgA*) [16–18]. However, a large number of other studies have shown an increased risk of gastric cancer in people with *cagA* positive *H. pylori* [19–21]*.* Recently, significant association between *vacA s1 am1* and *babA2* genotypes with cases of gastric ulcer and cancer has been reported in Saudi Arabia [22].

Furthermore, many studies across the world reported that the patients who are infected with *vacA s1* or *m1 H. pylori* strains have an increased risk of PUD and gastric carcinoma, in comparison to individuals infected with *s2*, *m2 H. pylori* strains [23, 24]. Moreover, another study has shown the role of *vacA s1* in the development of gastric carcinoma in the absence of *cagA* genes [25].

The main objectives of this study were to identify the distribution of *H. pylori cagA, vacA* and *iceA* genotypes among Southwestern Saudi dyspeptic patients, and to correlate them with the clinical outcomes and histopathological changes.

## Methods

### Patients and clinical specimens

Gastric biopsies were collected from 404 Saudi patients who attended gastroenterology clinics at general hospitals in Jazan (Southwestern region of Saudi Arabia). Gastric biopsies were obtained from each patient through the upper gastrointestinal endoscopy. The endoscopic examination findings were: gastritis, gastric ulcer, duodenal ulcer and gastric cancer.

### DNA extraction

There were non-sufficient biopsy materials in two cases, and from all the remaining 402 cases, DNA was extracted. First, the biopsy specimens were minced into small pieces by sterile scalpels, then the DNA was extracted by using DNeasy blood & tissue kit (Qiagen) according to manufacturer’s instructions.

### Real time (RT) PCR detection of *H. pylori*

All DNA samples extracted from the gastric biopsies (402 specimens) were submitted for quantitative real time PCR amplification using “genesig Quantification of *Helicobacter pylori*” kit (PrimerDesign Ltd. Southampton, United Kingdom) which is primer-probe based and targets the RNA polymerase beta-subunit (*rpo*B) gene of *H. pylori*. The reactions were carried in 20 μl volumes containing 10 μl of “oasig™ 2× qPCR Mastermix” (PrimerDesign Ltd.), 1 μl *H. pylori* specific primer/probe mix, 1 μl internal control primer/probe mix, 2 μl of internal control DNA, 3 μl of the sample extracted DNA, made up to a total volume of 20 μl by adding RNase/DNase free water supplied with the kit. The reactions were carried out using the Smart Cycler (Cepheid, Italy). Positive control (*H. pylori* DNA supplied with the kit) and negative control (contains RNase/ DNase free water) reactions were included in each PCR run. The PCR cycling conditions were 50 cycles of denaturation at 95 °C for 10 s and data collection at 60 °C for 60 s.

### Genotyping by detection of virulence genes of *H. pylori*

Overall, RT-PCR was positive in 187 specimens. The DNAs from these all 187 specimens were submitted for detection for *cagA*, *vacA* and *iceA* virulence genes. These genes were used for genotyping of the studied *H. pylori* strains.

Detection of these virulence genes was carried out by PCR using wide array of primers listed in Table 1. Each virulence gene was detected by a single PCR using its specified primers pair. All amplification reactions were carried out in total volumes of 50 μl containing reaction buffer 5 μl of 10× PCR buffer supplemented with MgCl_2_ (50 mM KCl, 10 mM Tris-HCl [pH 9.0], 15 mM MgCl_2_], deoxynucleoside triphosphate mix [0.2 mM each of dATP, dCTP, dGTP, and dTTP] (Roche, Germany), 0.4 μM of each primer, 2.5 U of FastStart *Taq* DNA polymerase (Roche, Germany), 5 μl of template DNA, made up to a total volume of 50 μl with sterile RNase and DNase free water. The PCR cycling conditions were 30 cycles of 94 °C for 10 min, 94 °C for 2 min, 55 °C for 2 min, and 72 °C for 2 min, followed by an extension step of 72 °C for 10 min. PCR was carried out using a thermal cycler (Eppendorf Mastercycler gradient, Germany). The resulting products were separated in 1.5% agarose gels and Tris-acetate-EDTA buffer (Promega, Madison, USA) and stained with ethidium bromide, and then images were captured using the Uvitec imaging system (Cambridge, UK).

**Table 1:** Primers used for genotyping of *H. pylori* by detection of *cagA*, *vacA* and *iceA* genes

Gene	Primer name	Primer sequence (5′ → 3′) ^a^	PCR product size	Reference
*cagA*	cagA-F	GATAACAGGCAAGCTTTTGAGG	349	[45]
	cagA-R	CTGCAAAAGATTGTTTGGCAGA		[45]
s1a	S1a-F	TCTYGCTTTAGTAGGAGC	212	[45]
	VA1-R	CTGCTTGAATGCGCCAAAC		[45]
s1b	SS3-R	AGCGCCATACCGCAAGAG	187	[45]
	VA1-R	CTGCTTGAATGCGCCAAAC		[45]
s1c	S1c-F	CTYGCTTTAGTRGGGYTA	213	[45]
	VA1-R	CTGCTTGAATGCGCCAAAC		[45]
s2	SS2-F	GCTAACACGCCAAATGATCC	199	[46]
	VA1-R	CTGCTTGAATGCGCCAAAC		[46]
m1	VA3-F	GGTCAAAATGCGGTCATGG	290	[47]
	VA3-R	CCATTGGTACCTGTAGAAAC		[47]
m2	VA4-F	GGAGCCCCAGGAAACATTG	352	[47]
	VA4-R	CATAACTAGCGCCTTGCAC		[47]
*iceA*1	iceA1-F	GTGTTTTTAACCAAAGTATC	247	[48]
	iceA1-R	CTATAGCCASTYTCTTTGCA		[48]
*iceA*2	iceA2-F	GTTGGGTATATCACAATTTAT	229/334	[48]
	iceA2-R	TTRCCCTATTTTCTAGTAGGT		[48]
*cag* empty site	Luni1	ACATTTTGGCTAAATAAACGCTG	550	[49]
	R5280	GGTTGCACGCATTTTCCCTTAATC		[49]

^a^Y is C or T, R is A or G and S is C or G

### Histopathological microscopic examination

All biopsies were submitted for histopathological examination to investigate the tissue inflammatory changes associated with infection. The biopsies were fixed in 10% formalin overnight, processed and embedded in paraffin wax. Four micron–thick tissue sections were taken and stained with the routine hematoxylin and eosin (H&E) stain, and modified Giemsa stain (Sheedhan’s modified method) [26] then examined according to the classification and grading system of Sydney [27].

### Statistical analysis

The SPSS version 20.0 (IBM Corp., NY, USA) was used for data analysis. Statistical analysis involved descriptive statistics as well as inferential statistics. Descriptive statistics included simple tabulation, frequencies and proportion for categorical variables including cross-tabulations. Differences in proportions were evaluated for significance using Chi Square/Fisher Exact test. *P*-values less than 0.05 were used to indicate statistical significance.

## Results

A total of 187 gastric biopsies were positive for presence of *H. pylori* by RT-PCR. Only 128 of them gave positive results in the genotyping PCR reactions.

### Genotyping PCR results of the three tested genes

**Fig. 1:**
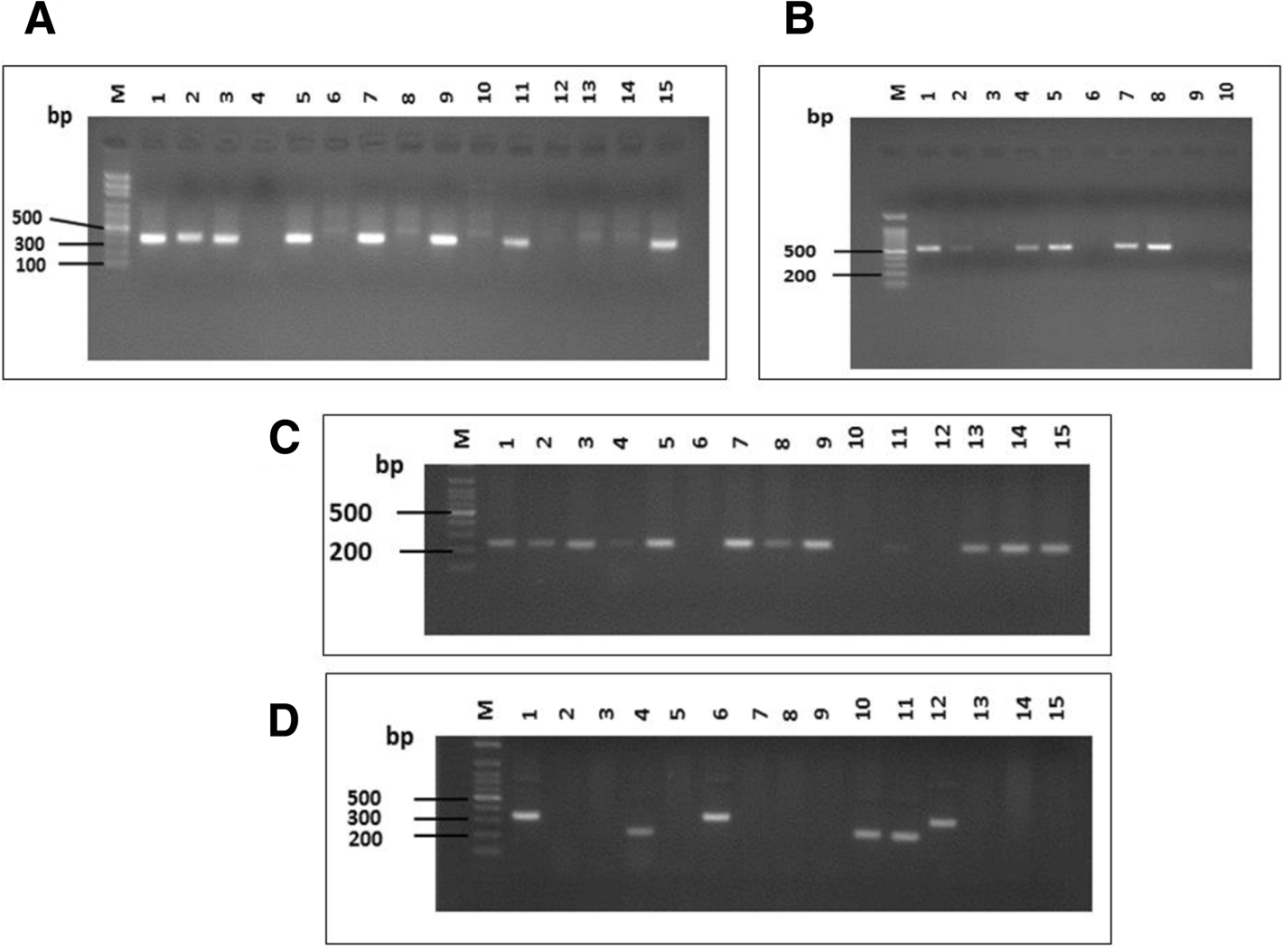
Agarose gel electrophoresis of the PCR-based genotyping amplified products of (**a**) *cagA* gene (349 bp). Lanes; M; 100 bp DNA ladder (Solis Biodyne). Lanes 1, 2, 3, 5, 7, 9, 11, and 15 are *cagA* positive while remaining lanes are negative for this gene. **b** *cag* empty site (550 bp). Lanes; M; 100 bp molecular DNA marker (Cleaver Scientific Ltd.). Lanes 1, 2, 4, 5, 7, 8 are *cagA* empty site positive while the remaining lanes are negative for this site. **c** *iceA*1 gene (247 bp). Lanes; M; 100 bp molecular DNA marker (Cleaver Scientific Ltd.). Lanes 1, 2, 3, 4, 5, 7, 8, 9, 11, 13, 14 and 15 are *iceA1* positive while remaining lanes are negative for this gene. Note that PCR bands in lanes 4 and 11 are faint. **d** *iceA*2 gene (229 or 334 bp). Lanes; M; 100 bp molecular DNA marker (Cleaver Scientific Ltd.). Lanes 1, 4, 6, 10, 11, and 12 are *iceA2* positive while lanes 2, 3, 5, 7–9, 13–15 are negative for this gene. Note that PCR products in lanes 1, 6 and 12 are of 334 bp size, while lanes 4, 10 and 11 have 229 bp PCR products

**Fig. 2:**
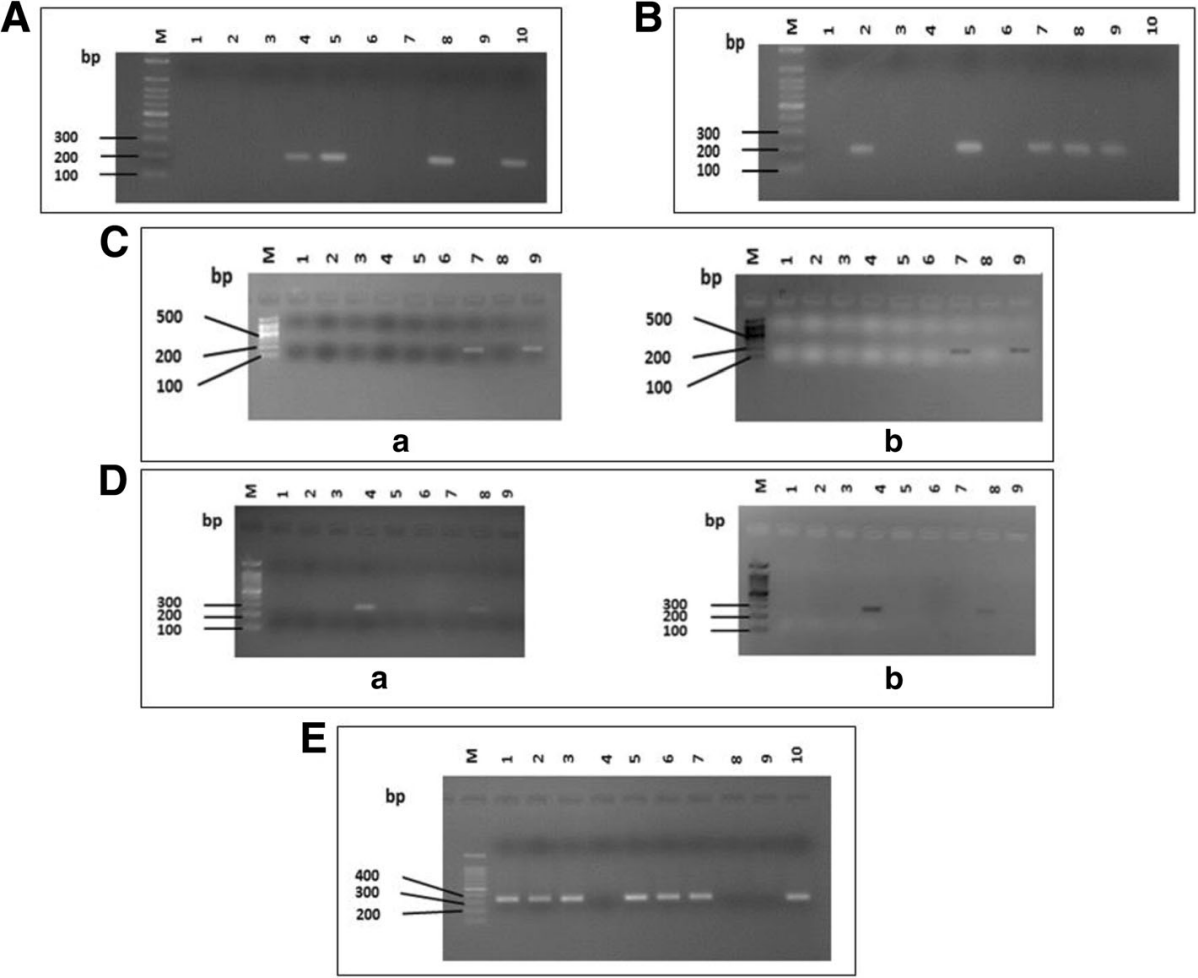
Agarose gel electrophoresis of the PCR-based genotyping amplified products of *vacA* gene. **a** PCR products of *vacA s1a* gene (212 bp). Lanes; M; 100 bp molecular DNA marker (Cleaver Scientific Ltd.). Lanes 4, 5, 8, 9, and 10 are positive while lanes 1, 2, 3, 6, 7 and 9 are negative. **b** PCR products of *vacA s1b* gene (187 bp). Lanes; M; 100 bp molecular DNA marker (Cleaver Scientific Ltd.). Lanes 2, 5, 7, 8 and 9, while lanes 10 are positive while lanes 1, 3, 4 and 10 are negative. **c** products of *vacA s2* gene (199 bp). a) Positive photo. b) Negative photo. Lanes; M; 100 bp molecular DNA marker (Cleaver Scientific Ltd.). Lanes 7 and 9 are positive while lanes 1, 2, 3, 4, 5, 6 and 8 are negative for this gene. **d** amplified products of *vacA m1* gene (290 bp). **a** Positive photo. **b** Negative photo. Lanes; M; 100 bp molecular DNA marker (Cleaver Scientific Ltd.). Lanes 4 and 8 are positive while lanes 1, 2, 3, 5, 6, 7 and 9 are negative for this gene. **e** products of *vacA m2* gene (352 bp). Lanes; M; 100 bp molecular DNA marker (Cleaver Scientific Ltd.). Lanes 1, 2, 3, 5, 6, 7 and 10 are positive while lanes 4, 8 and 9 are negative for this gene

We used two primer pairs to study the *cagA* gene status; one pair targeted *cagA* gene itself with production of amplified product of 349 bp size (Fig. 1a) and the other called *cag empty site* (550 bp in size) which is positive in cases negative for *cagA* gene (Fig. 1b). The PCR targeted *iceA*1 produced 247 bp-sized products (Fig. 1c); while the PCR amplified products of *iceA*2 gene were either 229 or 334 bp in size (Fig. 1d). Regarding *vacA* status (genotyping), the tested DNA samples were surveyed for s (signal) and m (middle) regions of *vacA* gene by multiple sets of primers targeted (*s1a*, *s1b*, *s1c* and *s2* alleles of *s* region, and *m1*, *m2* alleles of *m* region). The amplified products of these different genes are shown in Fig. 2. The specimens carried *s1* and *s2* or *m1* and *m2* were considered as mixed infection. The *s1a1b* was considered as a subtype of the *s1* genotype.

### Prevalence and distribution of *H. pylori cagA*, *iceA* and *vacA* genes

The results revealed that 49.2% (63/128) of tested *H. pylori* were *cagA* positive, 48.4% (62/128) were *cagA* negative and 2.3% (3/128) were mixed (positive for both *cagA* and *cagA* empty site genes). Regarding *iceA* gene: 42.2% (54/128) were *iceA*1 positive, 32.8% (42/128) were *iceA*2 positive, 3.9% (5/128) were *iceA*1 and *iceA*2 positive (mixed). Numerous subtypes of *vacA* gene were obtained as shown in Table 2. *s1as1bm2* subtype was the highest 23.4% (30/128), followed by *m2* and *s1a1b* subtypes which were equally detected 16.4% (21/128) for each. As regards the distribution of *vacA* genotypes according *cagA* status, *s1a1bm2* and *s1a1b* alleles were detected in higher levels in *cagA* positive subtypes, 31.7 and 27%, respectively. The distribution of *vacA* alleles according to *iceA* status revealed that *s1a1b* and *s1as1bm2* were highly detected in *iceA*1 subtypes (in rates of 20.4 and 18.5%, respectively), most of the *iceA*2 subtypes had *s1as1bm2, m2* and *s2 m2* alleles (28.6, 23.8, and 16.7%, respectively), while three of the *iceA* mixed five subtypes were having *s1as1bm2* alleles, and *s1as1b* allele was the mostly found allele in *iceA* negative subtypes (Table 2).

**Table 2:** Distribution of *H. pylori vacA* gene alleles according to *cagA* and *iceA* status

*vacA* alleles	*cagA*			*iceA*				Total^a^
	positive	negative	mixed	*iceA*1	*iceA*2	mixed	negative	
s1 am1	1	0	0	1	0	0	0	1
	1.6%	0.0%	0.0%	1.9%	0.0%	0.0%	0.0%	0.8%
s1bm1	1	0	0	0	0	0	1	1
	1.6%	0.0%	0.0%	0.0%	0.0%	0.0%	3.7%	0.8%
s1 am2	1	2	0	1	0	1	1	3
	1.6%	3.2%	0.0%	1.9%	0.0%	20.0%	3.7%	2.3%
s1bm2	7	4	0	5	3	0	3	11
	11.1%	6.5%	0.0%	9.3%	7.1%	0.0%	11.1%	8.6%
s2 m2	1	18	0	9	7	0	3	19
	1.6%	29.0%	0.0%	16.7%	16.7%	0.0%	11.1%	14.8%
s1a	7	2	0	5	1	0	3	9
	11.1%	3.2%	0.0%	9.3%	2.4%	0.0%	11.1%	7.0%
m2	3	18	0	7	10	1	3	21
	4.8%	29.0%	0.0%	13.0%	23.8%	20.0%	11.1%	16.4%
s1b	2	1	0	1	1	0	1	3
	3.2%	1.6%	0.0%	1.9%	2.4%	0.0%	3.7%	2.3%
s1a1b	17	4	0	11	3	0	7	21
	27.0%	6.5%	0.0%	20.4%	7.1%	0.0%	25.9%	16.4%
s1as1bm1	1	0	0	0	1	0	0	1
	1.6%	0.0%	0.0%	0.0%	2.4%	0.0%	0.0%	0.8%
s1as1bm2	20	8	2	10	12	3	5	30
	31.7%	12.9%	66.7%	18.5%	28.6%	60.0%	18.5%	23.4%
Mixed	1	3	1	2	3	0	0	5
	1.6%	4.8%	33.3%	3.7%	7.1%	0.0%	0.0%	3.9%
s2	0	2	0	1	1	0	0	2
	0.0%	3.2%	0.0%	1.9%	2.4%	0.0%	0.0%	1.6%
m1	1	0	0	1	0	0	0	1
	1.6%	0.0%	0.0%	1.9%	0.0%	0.0%	0.0%	0.8%
Total^b^	63	62	3	54	42	5	27	128
	100.0%	100.0%	100.0%	100.0%	100.0%	100.0%	100.0%	100.0%

^a^Total number of *vacA* alleles. ^b^ Total number of *vacA* alleles according to *cagA* or *iceA* status. *cagA* mixed genotypes contain both *cagA* and *cagA* empty sites amplified PCR products

### Distribution of genotypes according to the patients’ gender and age

Table 3 shows distribution of *H. pylori genes (vacA, cagA* and *iceA)* according to gender and age groups. Regarding the gender, overall, there was no significant difference in distribution of *vacA*, *cagA* and *iceA* genes between males and females. However, *vacA* subtype *m2* was higher among females (66.7% in females versus 33.3% in males). In contrast to that, *vacA s1a1b* was higher in males (61.9%), whereas in females it was 38.1%. As regards to patients’ age groups, *vacA m2* was higher (61.9%) in the young adults (age group between 13 and 29 years), while with an advance in age (age groups between 30 and 49 years and age groups above 50 years), the prevalence of this gene was decreasing (23.8 and 14.3%, respectively). However, no significant differences were found in distribution of other *vacA* subtypes, *cagA* and *iceA* genes among the different age groups.

**Table 3:** Distribution of genotypes according to the patients’ gender and age

Genotype/allele		Gender		*P*. value	Age groups			Total	*P. value*
		Male	Female		13–29	30–49	50+		
		N(%)	N(%)		N(%)	N(%)	N(%)		
*vacA*	s1 am1	0 (0.0)	1 (100.0)	0.514	0 (0.0)	1 (100.0)	0 (0.0)	1 (100.0)	0.305
	s1bm1	0 (0.0)	1 (100.0)		0 (0.0)	1 (100.0)	0 (0.0)	1 (100.0)	
	s1 am2	1 (33.3)	2 (66.7)		1 (33.3)	1 (33.3)	1 (33.3)	3 (100.0)	
	s1bm2	8 (72.7)	3 (27.3)		6 (54.5)	3 (27.3)	2 (18.2)	11 (100.0)	
	s2 m2	10 (52.6)	9 (47.4)		7 (36.8)	9 (47.4)	3 (15.8)	19 (100.0)	
	s1a	4 (44.4)	5 (55.6)		4 (44.4)	4 (44.4)	1 (11.1)	9 (100.0)	
	m2	7 (33.3)	14 (66.7)		13 (61.9)	5 (23.8)	3 (14.3)	21 (100.0)	
	s1b	2 (66.7)	1 (33.3)		1 (33.3)	2 (66.7)	0 (0.0)	3 (100.0)	
	s1a1b	13 (61.9)	8 (38.1)		3 (14.3)	12 (57.1)	6 (28.6)	21 (100.0)	
	s1as1bm1	0 (0.0)	1 (100.0)		0 (0.0)	0 (0.0)	1 (100.0)	1 (100.0)	
	s1as1bm2	17 (56.7)	13 (43.3)		7 (23.3)	12 (40.0)	11 (36.7)	30 (100.0)	
	mixed	1 (20.0)	4 (80.0)		3 (60.0)	2 (40.0)	0 (0.0)	5 (100.0)	
	s2	1 (50.0)	1 (50.0)		1 (50.0)	1 (50.0)	0 (0.0)	2 (100.0)	
	m1	0 (0.0)	1 (100.0)		0 (0.0)	1 (100.0)	0 (0.0)	1 (100.0)	
*cagA*	+ve	30 (47.6)	33 (52.4)	0.741	20 (31.7)	27 (42.9)	16 (25.4)	63 (100.0)	0.218
	-ve	32 (51.6)	30 (48.4)		26 (41.9)	24 (38.7)	12 (19.4)	62 (100.0)	
	mixed	2 (66.7)	1 (33.3)		0 (0.0)	3 (100.0)	0 (0.0)	3 (100.0)	
*iceA*	*iceA*1	27 (50.0)	27 (50.0)	0.528	16 (29.6)	28 (51.9)	10 (18.5)	54 (100.0)	0.571
	*iceA*2	19 (45.2)	23 (54.8)		18 (42.9)	15 (35.7)	9 (21.4)	42 (100.0)	
	mixed	4 (80.0)	1 (20.0)		2 (40.0)	1 (20.0)	2 (40.0)	5 (100.0)	
	negative	14 (51.9)	13 (48.1)		10 (37.0)	10 (37.0)	7 (26.0)	27 (100.0)	
Total		64 (50)	64 (50)		46 (35.9)	54 (42.2)	28 (21.9)	128 (100.0)	

### Association of *cagA*, *vacA* and *iceA* with clinical outcomes and histopathological changes

**Fig. 3:**
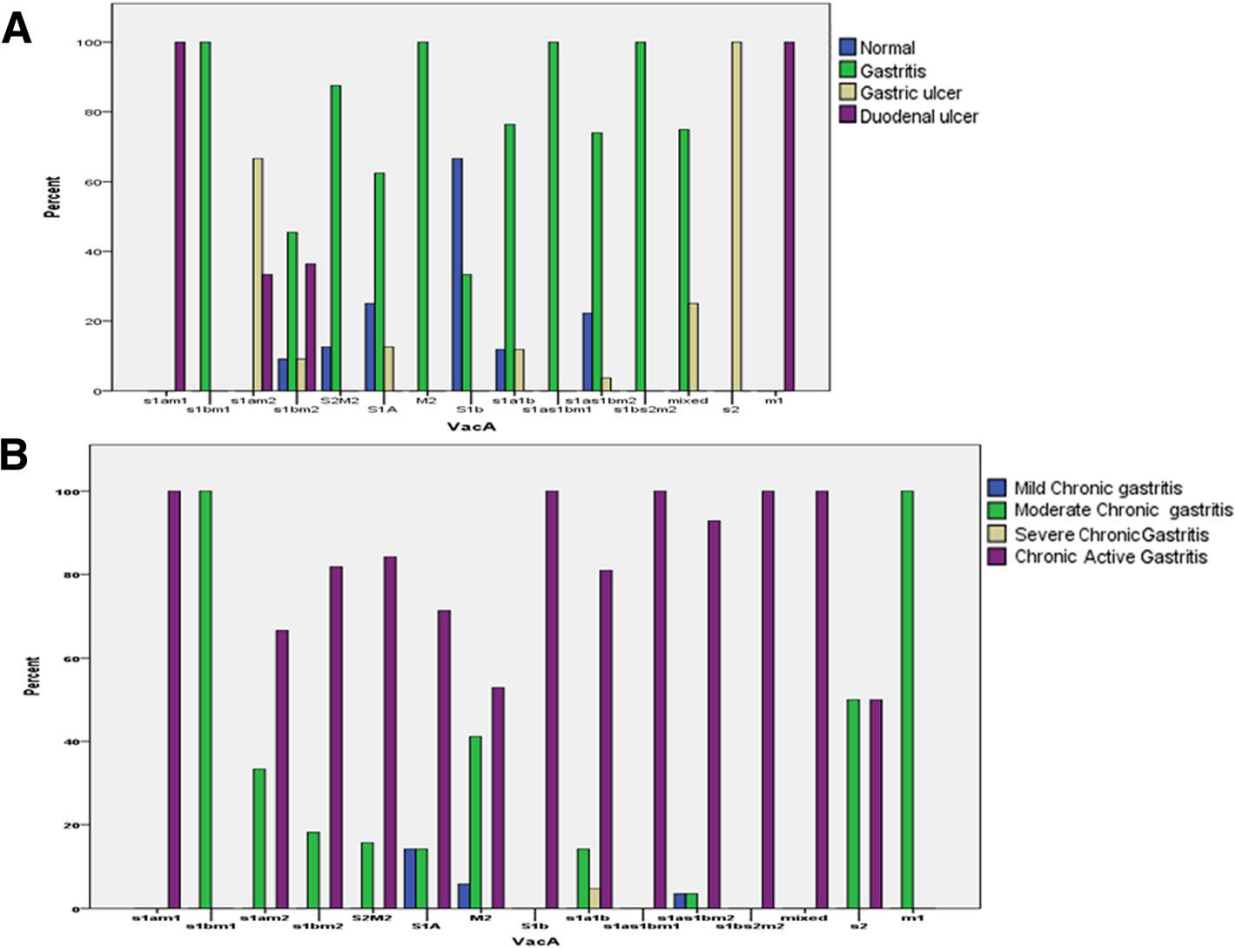
The relationship of *vacA* with: **a** clinical outcomes (as detected by endoscopic findings) and **b** histopathological changes

The clinical outcomes were assessed endoscopically as normal, gastritis, gastric ulcer and duodenal ulcers, while the histopathological changes were determined by histopathological examination and were classified into: mild chronic gastritis, moderate chronic gastritis, severe gastritis, chromic active gastritis. The relationship between *H. pylori* genes (*cagA*, *iceA* and *vacA*) and the clinical outcomes (endoscopic findings) and histopathological findings are presented in Table 4 and Fig. 3.

**Table 4:** Distribution of *H. pylori cagA* and *iceA* genotypes according to endoscopic findings and histopathological changes

	*cagA* status N(%)			*P*. value	*iceA* status N(%)				Total	*P*. value
	positive	negative	mixed		*iceA*1	*iceA*2	mixed	negative		
Endoscopic findings										
Normal	9 (45.0)	11 (55.0)	0 (0.0)	0.480	4 (20.0)	6 (30.0)	0 (0.0)	10 (50.0)	20 (100.0)	0.026
Gastritis	44 (48.4)	44 (48.4)	3 (3.3)		42 (46.2)	31 (34.1)	4 (4.4)	14 (15.4)	91 (100.0)	
Gastric ulcer	4 (40.0)	6 (60.0)	0 (0.0)		6 (60.0)	3 (30.0)	1 (10.0)	0 (0.0)	10 (100.0)	
Duodenal ulcer	6 (85.7)	1 (14.3)	0 (0.0)		2 (28.6)	2 (28.6)	0 (0.0)	3 (42.9)	7 (100.0)	
Histopathological changes										
Mild chronic gastritis	3 (60.0)	2 (40.0)	0 (0.0)	0.724	2 (40.0)	3 (60.0)	0 (0.0)	0 (0.0)	5 (100.0)	0 .479
Moderate chronic gastritis	11 (44.0)	14 (56.0)	0 (0.0)		13 (52.0)	6 (24.0)	2 (8.0)	4 (16.0)	25 (100.0)	
Severe gastritis	2 (100.0)	0 (0.0)	0 (0.0)		2 (100.0)	0 (0.0)	0 (0.0)	0 (0.0)	2 (100.0)	
Chromic active gastritis	47 (49.0)	46 (47.9)	3 (3.1)		37 (38.5)	33 (34.4)	3 (3.1)	23 (24.0)	96 (100.0)	
Total	63 (49.2)	62 (48.4)	3 (2.3)		54 (42.2)	42 (32.8)	5 (3.9)	27 (21.1)	128 (100.0)

Although no significant differences of *cagA* status according to clinical outcome or histopathological changes, more than 80% of duodenal ulcers were *cagA* positive and all 3 mixed *cagA* genotypes were positive for chronic active gastritis. The *iceA*1and *iceA*2 genes were significantly associated with gastritis and gastric ulcer (*p*-value of 0.026) and there was an apparent association between *iceA*1 and *iceA*2 genes and chronic active gastritis with percentages of 38.5 and 34.4%, respectively, in comparison to *iceA* negative (24.0%). Moreover, all mixed *iceA* cases showed gastritis either moderate chronic or chronic active.

The prevalence of *H pylori* genes *vacA* according endoscopic findings and histopathological changes is shown in Fig. 3. Overall, *vacA* genotype was significantly associated with clinical outcomes (*p*-value of 0.000), as *vacA* subtypes: *s1a1b*, *s1as1bm2*, *s2 m2* and *m2*, *s1bm2* were significantly correlated to gastritis, whereas, subtypes *s1 am1*, *s1 am2*, m1were significantly associated with gastric and duodenal ulcers. The subtype *vacA s1bm2* was associated with gastritis in 5 cases (45.5%) and with duodenal ulcer in 4 cases (36.4%). Eighty percent of mixed *vacA* genotypes showed gastritis. Regarding the association between *vacA* genotype and the histopathological changes, although there was no statistical significance (*p*- value of 0.232), *vacA* subtypes: *s1as1bm2*, *s1a1b* and *s2 m2* showed chronic active gastritis in percentages of 90.0, 81, 84.2%, respectively. The subtype *vacA s1bm2* was associated mainly with chronic active gastritis (9 cases, 81.8%), while the subtype *vacA m2* was presented with chronic active gastritis in 10 (47.6%) cases and with moderate active gastritis in 9 (42.9%) cases. All *vacA* mixed genotypes showed chronic active gastritis.

## Discussion

Previous studies on the association of *H. pylori* genotypes with the clinical outcomes in Saudi Arabia are so scarce [22]. Our data revealed that the overall prevalence of *cagA* gene was 49.2% (63/128). Regarding the distributions of *cagA* gene in relation to endoscopic and histopathological findings, more than 80% of duodenal ulcer cases were *cagA* positive and all the 3 *cagA* mixed types were positive in cases of chronic active gastritis. However, no statistical significance was found. The distribution of *cagA* genes and its association with the clinical outcomes in our study is consistent with the previous studies on *H. pylori* genes in Saudi Arabia by Momenah et al*,* Marie et al.and Kadi et al.*,* in which, they found that the overall prevalence of *cagA* was 52.4, 62 and 81.8%, respectively [10, 14, 28]. In agreement with our findings, Momenah et al. and Marie et al. found an association between *cagA* gene and peptic ulcers (100 and 71%, respectively). However, Kadi et al. revealed no significant association. In contrast to our findings, Seriki et al and colleagues reported a high prevalence of *cagA* gene among the studied patients (100%). As similar to our findings, they found no association of stastistical significance between *cagA* genes and clinical outcomes [29]. On the other hand, a recent study from Brazil by Sallas et al. found *cagA* gene in 50% of *H.pylori* isolates [30]^.^ This percentage is consistent with ours. Also, similar to our data another study from Ecuador reported *cagA* prevalence rate of 45.9% [30, 31]. In contrary to our results Sallas et al. [30] found no correlation between *cagA* gene alone and clinical outcomes. However, they found significant asscoiation between *cagA/vacA* genes and chronic gastritis and gastric cancer. Our results are different from a recent Mexican study by Cantu et al. and colleagues, who investigated for *H.pylori* genotypes in oral cavities of 100 asymptomatic children [32]. They reproted *cagA* prevalence rate of 80.8% among the studied subjects. This difference may be due to variations in study population, ages and the site of sample collection. However, in agreement with our data, they found correlation between *cagA* status and clinical outcomes. More recently, an Egyptian study by Abu-Taleb et al. reported *cagA* prevalence of 57.4% in the studied subjects [33]. In other parts of the world, the rates of *H. pylori cagA* genes were 90% in East Asia (Japan and Korea) and 60% in North America, Europe and Cuba [34]. The wide variation of *cagA* prevalence rates a cross the globe could be attributed to the differences in: study sizes, socioeconomic, geographical as well as genetic factors.

In the present study, *vacA* gene was detected in all the isolates (100%). Similar to our results, El Khadir et al. found *vacA* detection rate of 99% in Morocco, whereas low rates of *vacA* gene (90, 93%) were reported in Ethiopia and Netherland, respectively [35]. In the current study, the most predominant *vacA* subtype were *s1as1bm2* (23.4%), *m2* (16.4%) and *s1a1b* (16.4%). Whereas, *s2 m2* subtype was found in 14.8% (19/128). On the other hand, our data showed that: *s1 am1, s1bm1* and *m1* were the least *vacA* subtypes (representing 0.8%). In agreement with our findings, Pajavand et al. [9] reported that the predominant *vacA* subtypes were *s1 m2* and *s2 m2* with frequency of 39.5 and 50%, respectively, whereas the least subtype was *s1 m1* with frequency of only 7%. However, *vacAs1 m1 and vacAs1 m2* were the predominat according to Cantu et al. report [32]. Furthermore, Marie et al. [10] investigated *vacA* genes in Saudi population. He reported frequencies as follows: *vacA s1 m1* (28%), *s1 m2* (40%) and *s2 m2* (26%) in subjects with peptic ulcer and gastritis. Our results were consistent with his findings regarding distribution of *vacA s1 m2* and *s2 m2* subtypes. In contrast to Marie et al. [10], Sallas et al. [30] and Cantu et al. [32] results, we found very low *s1 am1* rate in the *H. pylori* + ve strains. The low *vacA s1 m1* in the present study could be attributed to the differences in: study sizes, regions and population.

It has been well established that *vacA s1/m1* is associated with severe damage to the gastric epithelium [36]. Worldwide, numerous studies had reported *vacAs1 m1* frequency of 24–84% [9].

With respect to the association of these *vacA* subtypes with the clinical outcomes, our results revealed a significant association (with *p*-value of 0.000 for all). In addition, *vacA s1 am1*,*s1 am2*, *s1bm2* were the predominant in gastric ulcer and duodenal ulcer. These findings were in agreement with Sallas et al. [35] and Pajavand et al. [9] results. A recent study from Saudi Arabia, showed high *vacA s1* in subjects with gastric ulcer and gastric cancer (80 and 100%, respectively) [22]. Several studies from China, Middle East, Africa and Western countries have shown that individuals infected with *vacA* s1 or *m1 H. pylori* strains have an increased risk of peptic ulcer or gastric cancer compared with individuals infected with *s2 or m2* strains, so *s2 and m2* strains are considered as less virulent [24, 37, 38]. Surprisingly, Sedaghat et al. [8] found no significant correlation between *vacA* genes and clinical outcomes. In the present study we detected high rate of *vacAs1a1b* (16%).This finding is inconsistent with the previous studies. Interestingly, *vacAs1a1b* was found to be significantly associated with chronic active gastritis. It is well-known that chronic active gastritis can evolve into atrophic gastritis, intestinal metaplasia, dysplasia, which may eventually lead to the development of gastric cancer [39].

In the current study, mixed *vacA*, *cagA* and *iceA* genotypes were seen in 3.9%, 2.3, 3.9% of cases. The rates of mixed genotypes vary from 0 to 85% in different populations across the world [40]. In the present study, we found correlation between *cagA* positive strains and the most virulent *vacA* subtypes such as *s1a1bm2* and *s1a1b*, whereas in *cagA* negative strains, the less virulent subtypes such *s2 m2* and *m2* were the most predominant. Boukharis et al. found a significant association between *vacA s1 m1* and intestinal metaplasia [41]. Also, Matsunari et al. found that the association of *cagA* strains with *vacA s1* increase the risk of atrophic gastritis and gastric cancer [42]. However, Hussein et al revealed no correlation between *H. pylori* virulence genes (*vacA*, *cagA* and *dupA*) and histopathological changes in Iraqi patients [36].

With respect to *iceA* genotype/subtypes, our results revealed that 42.2% of *H. pylori* were harboring *iceA*1, 32.8% were *iceA*2, whereas 3.9% were mixed (*iceA*1/*iceA*2). Similar to our findings Abu-Taleb et al. [33] and Sedaghat et al. [8] reported *iceA* gene prevalence rates of 46.29 and 48.6%, respectively. Our results showed a significant association of *iceA*1 and *iceA*2 genes with gastritis and gastric ulcer (*p*-value of 0.026). Furthermore, there was a clear association between *iceA*1, *iceA*2 and chronic active gastritis (38.5, 34.4%, respectively), although it was not statistically significant. Kadi et al. [14], Momenah et al. [28], and Abu-Taleb et al. [33] were in agreement with our data regarding the correlation of *iceA*1 with gastritis and peptic ulcer. Also, numerous studies have shown a similar association between *iceA* genes and the clinical outcomes [43]. In contrast to our results, some studies from Brazil and Iran showed no association between *iceA1* gene and gastritis [8, 44].

As similar to other studies, our results revealed no gender differences in terms of distribution of *H. pylori cagA* and *iceA* genotypes [14]. However, some *vacA* subtypes revealed gender variations in the present study. We found high rates of the less virulent *vacA m2* among females (65%), compared to 35% in males. Whereas, *vacA s1a1b* was predominant in males (61.9%), compared to 38.1% in females. In concordance with our results, El Khadir et al. found that *vacA* subtypes (*s1, i1 and m1*) were more prevalent in males than in females [35]. In contrary to our results, he found no differences in relation to age. However, we found that *vacA m2* gene was higher (61.9%) in young adults (age group between 13 and 29 years). While, in older age groups, the prevalence of this gene was decreasing (23.8 and 14.3% in the age group between 30 and 49 years and above 50 years, respectively). Similar observations were reported by Feliciano et al. [34].

## Conclusions

This study showed a high prevalence and diversity of *H. pylori* virulence genes among patients with dyspepsia in Southwestern region of Saudi Arabia. There was a significant association between different *vacA* subtypes and *iceA* genes with the clinical outcomes. Moreover, there were some variations in the distribution of virulence genes with respect to age and gender. Furthermore, there was a significant association between some *vacA* subtypes, *iceA* genes and histopathological changes.

## Abbreviations

Bp: Base pair
*cagA*: cytotoxin-associated gene A
dATP: Deoxyadenosine triphosphate
dCTP: Deoxycytidine triphosphate
dGTP: Deoxyguanosine triphosphate^[^
DNA: Deoxyribonucleic acid
DNase: Deoxyribonuclease enzyme
dTTP: Deoxythymidine triphosphate
EDTA: Ethylenediaminetetraacetic acid
H&E: Hematoxylin and eosin
*H. pylori*: *Helicobacter pylori bacteria*
PCR: Polymerase chain reaction
PUD: peptic ulcer disease
RNase: Ribonuclease enzyme
RT-PCR: Real time polymerase chain reaction
*vacA*: Vacuolating cytotoxin gene A
WHO: World Health Organsation
